# Supplementary material for: The Construction and Exploration of a Comprehensive MicroRNA Centered Regulatory Network in Foxtail Millet (Setaria italica L.)
Source: Front Plant Sci. 2022 May 6;13:848474. doi: 10.3389/fpls.2022.848474 (PMC9121102; doi:10.3389/fpls.2022.848474)
Supplement: Supplementary file 7 [file Data_Sheet_7.zip › Sit-miRN3001b_Seita.5G081800.1_374_TPlot.pdf]

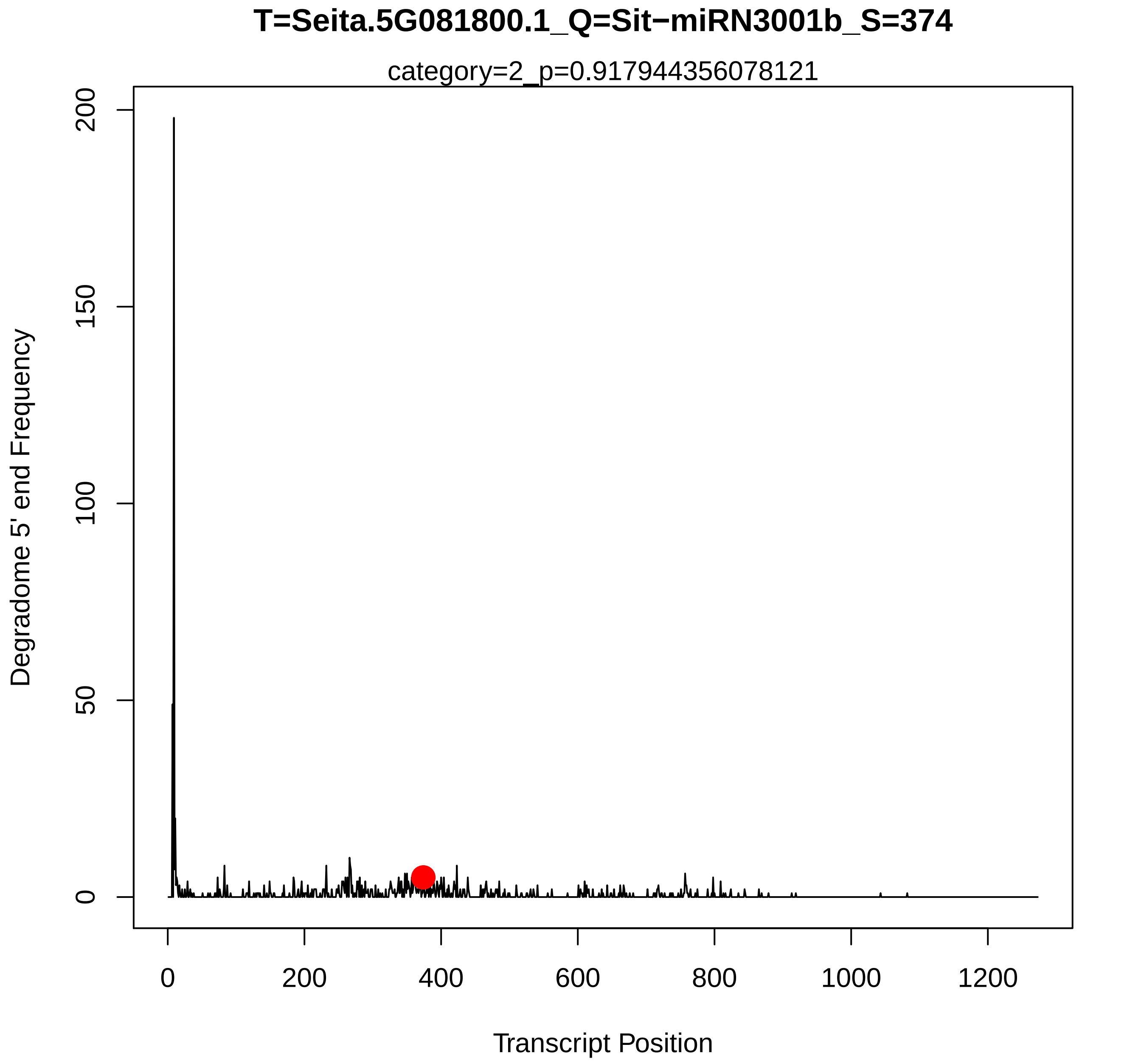

T=Seita.5G081800.1_Q=Sit−miRN3001b_S=374
category=2_p=0.917944356078121
Degradome 5' end Frequency
Transcript Position
0
50
100
150
200
0
200
400
600
800
1000
1200
